# Alterations in the Synthesis of IL-1β, TNF-α, IL-6, and Their Downstream Targets RANKL and OPG by Mouse Calvarial Osteoblasts *In vitro*: Inhibition of Bone Resorption by Cyclic Mechanical Strain

**DOI:** 10.3389/fendo.2013.00160

**Published:** 2013-10-28

**Authors:** Salvador García-López, Rosina Villanueva, Murray C. Meikle

**Affiliations:** ^1^Health Science Department/Cell Biology and Immunology Laboratory, Universidad Autónoma Metropolitana-Xochimilco, Mexico City, Mexico; ^2^Orthodontic Department, General Hospital “Dr. Manuel Gea González”, Universidad Nacional Autónoma de México, Mexico City, Mexico; ^3^Orthodontic Department, Universidad Intercontinental, Mexico City, Mexico; ^4^Faculty of Dentistry, National University of Singapore, Singapore

**Keywords:** mouse osteoblasts, mechanical deformation, pleiotropic cytokines, RANKL, OPG

## Abstract

Mechanical strain is an important determinant of bone mass and architecture, and the aim of this investigation was to further understand the role of the cell–cell signaling molecules, IL-1β, TNF-α, and IL-6 in the mechanobiology of bone. Mouse calvarial osteoblasts in monolayer culture were subjected to a cyclic out-of-plane deformation of 0.69% for 6 s, every 90 s for 2–48 h, and the levels of each cytokine plus their downstream targets RANKL and OPG measured in culture supernatants by ELISAs. Mouse osteoblasts constitutively synthesized IL-1β, TNF-α, and IL-6, the production of which was significantly up-regulated in all three by cyclic mechanical strain. RANKL and OPG were also constitutively synthesized; mechanical deformation however, resulted in a down-regulation of RANKL and an up-regulation OPG synthesis. We next tested whether the immunoreactive RANKL and OPG were biologically active in an isolated osteoclast resorption pit assay – this showed that culture supernatants from mechanically deformed cells significantly inhibited osteoclast-mediated resorptive activity across the 48 h time-course. These findings are counterintuitive, because IL-1β, TNF-α, and IL-6 have well-established reputations as bone resorptive agents. Nevertheless, they are pleiotropic molecules with multiple biological activities, underlining the complexity of the biological response of osteoblasts to mechanical deformation, and the need to understand cell–cell signaling in terms of cytokine networks. It is also important to recognize that osteoblasts cultured *in vitro* are deprived of the mechanical stimuli to which they are exposed *in vivo* – in other words, the cells are in a physiological default state that in the intact skeleton leads to decreased bone strains below the critical threshold required to maintain normal bone structure.

## Introduction

Mechanical stimuli play an important role in the growth, structure, and maintenance of skeletal tissues. It has been estimated that environmental factors such as physical activity and nutrition account for 20–40% of individual variation in bone mass, the remaining 60–80% being determined by genetic factors (1, 2). Mechanical stimuli may be growth-generated as in embryonic tissues with differential growth rates (3), the result of functional movement as in synovial joints (4, 5), the consequence of physical activity (6), or by the activation of orthodontic appliances. In contrast, prolonged bed rest or weightlessness leads to bone loss and osteopenia (7, 8).

In the adult skeleton, during normal physiological turnover there is a balance between the amount of bone resorbed by osteoclasts and that formed by osteoblasts to maintain a constant bone mass (9). Bone resorption and bone formation are therefore said to be coupled, a process of renewing the skeleton while maintaining its structural integrity, embodied in the A-R-F (activation-resorption-formation) sequence of the bone remodeling cycle. Bone remodeling is orchestrated by cells of the osteoblast lineage and involves a complex network of cell–cell signaling mediated by systemic osteotropic hormones, locally produced cytokines, growth factors, and the mechanical environment of the cells (10–13). One of the most significant developments in connective tissue biology during the 1980s was the finding that cytokines such as interleukin-1 (IL-1), tumor necrosis factor (TNF), and IL-6, originally identified as immunoregulatory molecules, could also act as regulators of pathophysiological resorption (14–17) and were produced by many different cell types including osteoblasts (18).

Another key advance was the observation that osteoclast formation and function *in vitro* was dependent upon the presence of stromal cells/osteoblasts, which suggested that soluble factor(s) were involved in osteoblast–osteoclast signaling (19). This led to the discovery of OPG (osteoprotegerin) and RANKL (receptor activator of nuclear factor κB ligand), two cytokines synthesized by osteoblasts (20–24), and constituents of a ligand–receptor system known as the RANK/RANKL/OPG triad that directly regulates the final steps of the bone resorptive cascade. RANKL which exists in both membrane-bound and soluble forms stimulates the differentiation and function of osteoclasts, an effect mediated by RANK, a member of the TNF receptor family expressed primarily on cells of the monocyte/macrophage lineage, including osteoclasts and their precursor cells (25). OPG is a secreted protein that inhibits osteoclastogenesis by acting as a decoy receptor, binding to and neutralizing both cell-bound and soluble(s) RANKL.

Following the initial mechanotransduction event at the cell membrane, mechanical stimuli appear to influence bone remodeling by their ability to regulate the synthesis and/or action of cytokines. Since remodeling occurs at distinct sites throughout the skeleton, osteoblast cytokines are ideally placed to regulate or modify the action of other cell types in bone, although the interactions are complex and poorly understood. Using mouse calvarial osteoblasts as our model, the aim of this study was to determine the effect of cyclic mechanical strain on the synthesis and biological activity of the pleiotropic cytokines IL-1β, TNF-α, IL-6, and their downstream targets RANKL and OPG.

## Materials and Methods

### Preparation of mouse osteoblasts

Calvarial osteoblasts were prepared and characterized by a modification of the method previously described by Heath et al. (26). Neonatal mouse calvaria from BALB/C mice were dissected free from adherent soft tissue, washed in Ca^2±^ and Mg^2±^free Tyrode’s solution (10 min) and sequentially digested with 1 mg/ml trypsin (for 20 and 40 min). Cells from these digests were discarded; the bones were washed in phosphate buffered saline (PBS) and cut into pieces for a third trypsin digest (20 min). The cells released from this digest were washed in PBS, centrifuged at 1000 rpm for 5 min and the pelleted cells resuspended in 1:1 F12/Dulbecco’s modification of Eagle’s medium (DMEM) supplemented with 20% fetal calf serum (GIBCO, Invitrogen, Carlsbad, CA, USA), 100 units/ml penicillin, and 100 μg/ml streptomycin, then seeded into 75-cm flasks and grown to confluence at 37°C in a humidified atmosphere of 5% CO_2_/95% air. The cells were identified as osteoblasts by morphological criteria and the fact that more than 95% stained strongly for alkaline phosphatase (ALP).

### Application of mechanical deformation to mouse osteoblasts

After the cells had reached confluence (20–25 days), adherent cells were detached with trypsin-EDTA (0.25%; Sigma), resuspended in F12/DMEM with 10% fetal calf serum (Gibco), 100 units/ml penicillin and 100 μg/ml streptomycin and plated at an initial cell density of 10^6^ cells/dish into 35 mm Petriperm dishes (*In vitro* Systems & Services GmbH, Germany) with flexible bases. Vacuum pressure was used to displace the substrate – maximal deflection 2 mm, according to the method of Banes et al. (27) and a cyclic strain applied to the cells for 6 s (0.166 Hz), every 90 s for 2–48 h as described previously (28). The maximal strain applied to the cells was calculated according to the formula:

$$Arc=\frac{1}{2}\sqrt{d^{2}+16\;b^{2}}+\frac{d^{2}}{8b}\;1n\left(4b+\frac{\sqrt{d^{2}+16\;b^{2}}}{d}\right)$$

*d* = diameter (33 mm); *b* = maximum deflection (2 mm); *Arc* = 33.23 mm

$$\max\;\mathrm{strain}=\frac{Arc-d}{d}100=0.69\%.$$

Each dish contained 4 ml of F12/DMEM medium; 500 μl was sampled at each time point and 500 μl fresh medium added. Because the deformation is out-of-plane, the level of strain experienced by the cells will be greatest at the center and least at the perimeter of the substrate and roughly half that programed into the computer. The overall level of deformation is therefore comparable with strain levels recorded at the surface of diaphyseal bone *in vivo* (1–3 × 10^6^ microstrain) depending on location following dynamic loading (29, 30).

### Culture media proteomics

Media samples were supplemented with 1 mg/ml protease inhibitor cocktail (Sigma-Aldrich P1860, St. Louis, MO, USA), stored at −70°C and assayed 2 days later for IL-1β, TNF-α, IL-6, OPG, and soluble sRANKL protein by enzyme-linked immunosorbent assays (ELISAs; R & D Systems, Minneapolis, MN, USA). Absorbance was measured at 450 nm according to the manufacturer’s instructions.

### Osteoclast resorption pit assay

The osteoclast resorption assay is based on the ability of isolated osteoclasts to resorb cortical bone, dentine, or ivory slices *in vitro* (31). Ivory was chosen as the substrate being free of vascular channels and pre-existing resorbing surfaces and osteoclasts produce resorption pits in its smooth surface greatly facilitating quantification. Ivory slices (250 μm in thickness) were cut with a Micro Slice 2 machine (Metals Research, Cambridge, England) at low speed from a 1 cm diameter rod. Osteoclasts were obtained from the femurs of 2–3-day-old BALB/C mice and allowed to settle on the slices for 20 min at 37°C as described previously (32). The substrate was then washed free of non-adherent cells, and the slices incubated for 24 h in a humidified atmosphere of 5% CO_2_/95% air at 37°C in 500 μl of conditioned medium plus 500 μl of fresh DMEM supplemented with 5% fetal calf serum, 100 units/ml penicillin, and 100 μg/ml streptomycin in 1.5 cm multiwall plates. At the completion of the culture period the cells were removed, the ivory slices stained with trypan blue and resorption quantified by measuring the surface area of the resorption lacunae by image analysis (Stereoscopy Microscope model SKD/SKO/KTD, Arhe, Holland). A single experiment consisted of eight ivory slices bearing the cells from one mouse, with four slices for each control and test variable.

### Statistical analysis

Data are expressed as mean ± standard error of the mean (SEM). Differences between control and experimental cultures were determined by the Student’s *t*-test (two tailed) using GraphPad Prism 4 software (GraphPad Software Inc., San Diego, CA, USA) and the level of significance set at *P* < 0.05.

## Results

### Effects of cyclic mechanical strain on cytokine production

**Figure 1:**
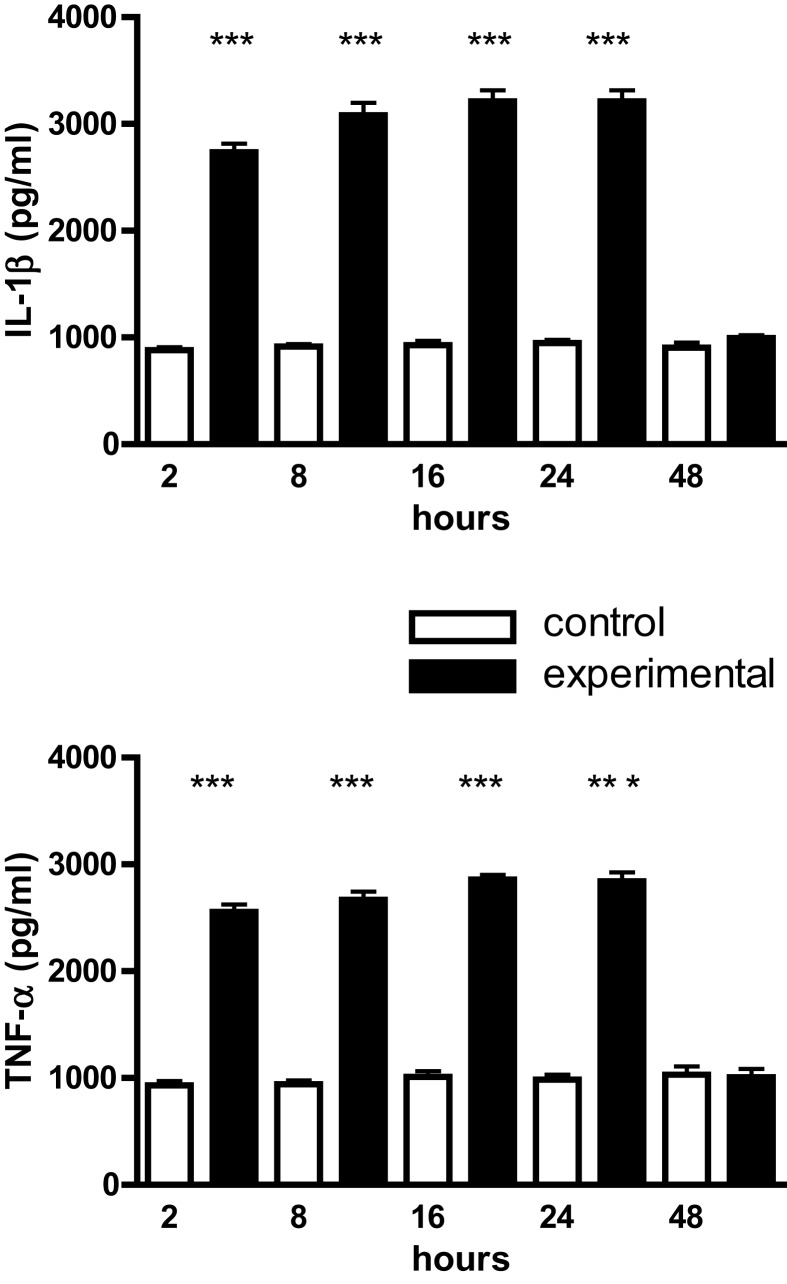
**IL-1β and TNF-α production by mouse calvarial osteoblasts**. Osteoblasts in monolayer culture were subjected to a cyclic tensile strain (6 s every 90 s) for 2–48 h and the culture media assayed for IL-1β and TNF-α by ELISAs. Results are expressed as mean ± SEM for 10 cultures. ***Experimental significantly greater than control. *P* < 0.001.

**Figure 2:**
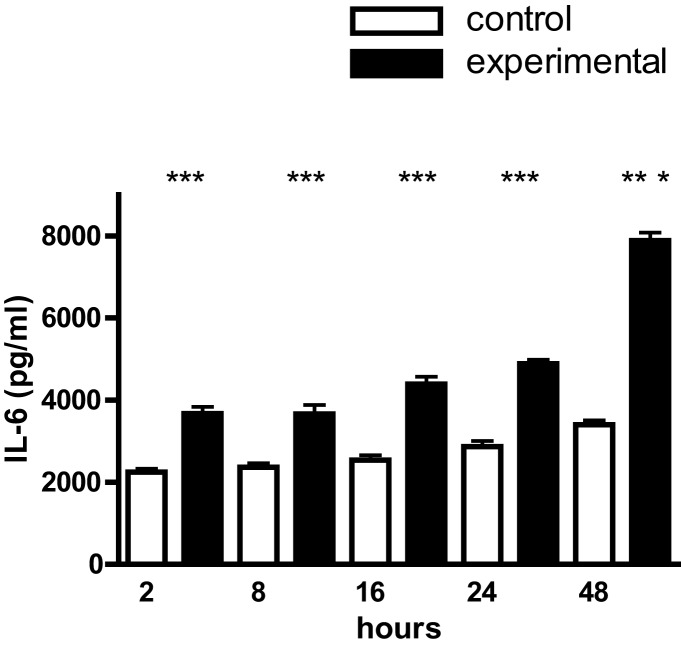
**IL-6 production by mouse calvarial osteoblasts**. Osteoblasts in monolayer culture were subjected to a cyclic tensile strain (6 s every 90 s) for 2–48 h and the culture media assayed for IL-6 by an ELISA. Results are mean ± SEM for 10 cultures. ***Experimental significantly greater than control.*P* < 0.001.

Mouse calvarial osteoblasts in monolayer culture constitutively synthesized IL-1β, TNF-α, and IL-6 over the 48 h time-course of the experiments; for IL-1β and TNF-α the levels were 10^3^ pg/ml and for IL-6, 2–3 × 10^3^ pg/ml (Figure 1). Cyclic tensile strain significantly up-regulated IL-1β and TNF-α synthesis two- to threefold from 2 to 24 h, returning to control levels by 48 h (Figure 1). In the case of IL-6 the increments were smaller (one- to twofold), but of greater magnitude (4–8 × 10^3^ pg/ml), and were sustained over the entire 48 h time-course (Figure 2).

### Effects of cyclic strain on sRANKL and OPG

**Figure 3:**
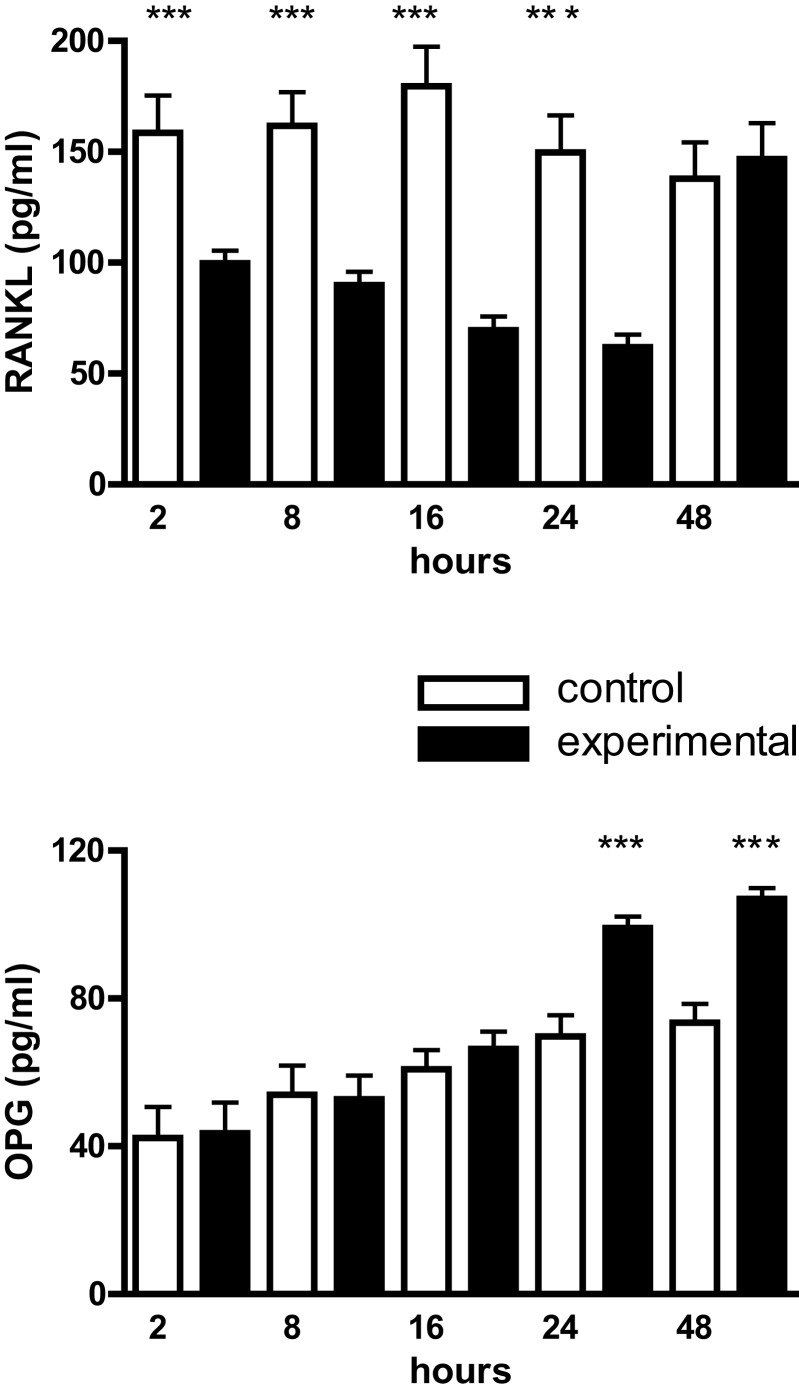
**RANKL and OPG production by mouse calvarial osteoblasts**. Osteoblasts in monolayer culture were subjected to a cyclic tensile strain (6 s every 90 s) for 2–48 h and the culture media assayed for RANKL and OPG by ELISAs. Results are expressed as mean ± SEM for 10 cultures. ***Experimental significantly different from control.*P* < 0.001.

Cultured mouse osteoblasts constitutively synthesized sRANKL and OPG. From 2 to 24 h there was a significant reduction in the level of sRANKL of approximately one- to twofold in mechanically deformed cultures; from 24 to 48 h, however, immunoreactive sRANKL returned to control levels (Figure 3). In contrast, OPG levels were not significantly different over the first 24 h, but from 24 to 48 h had increased by approximately 50% in culture media from mechanically deformed cells (Figure 3).

### Inhibition of osteoclast resorption

**Figure 4:**
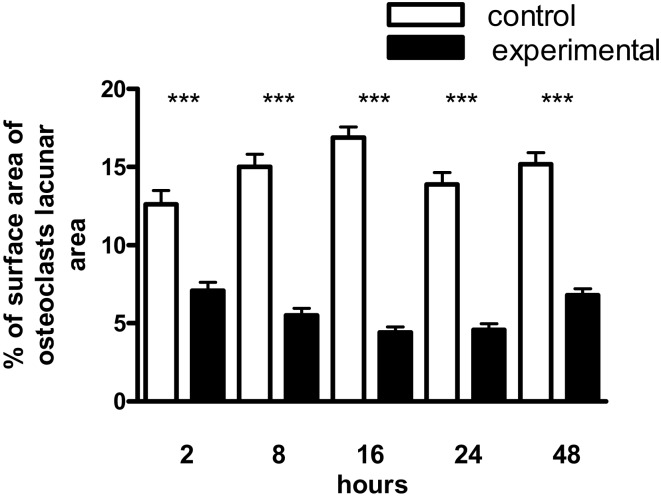
**Effect of conditioned media from osteoblast cultures on the surface area of mouse osteoclast resorption lacunae**. Osteoclasts were obtained from the femurs of 2–3-day-old BALB/C mice and allowed to settle on ivory slices for 20 min at 37°C. The substrate was washed free of non-adherent cells and the slices incubated for 24 h in 500 μL of conditioned medium plus 500 μl fresh DMEM; resorption was quantified by measuring the surface area of the resorption lacunae by image analysis. The values represent the means ± SEM from four slices at each time point. ***Experimental significantly less than control.*P* < 0.001.

In view of the well-established ability of IL-1β, TNF-α, and IL-6 to stimulate bone resorption *in vitro*, and the importance of OPG and RANKL in regulating the terminal pathway of the bone resorptive cascade, we next tested the biological activity of the RANKL/OPG ratio in the culture media using an isolated osteoclast resorption pit assay. Figure 4 shows the contrary to expectation there was a significant inhibition of osteoclast resorption by culture media from mechanically strained cultures over the entire 2–48 h time scale.

## Discussion

Rubin et al. (33) have shown that tensile mechanical strain (2% at 10 cycles/min) applied to mouse bone marrow stromal cells *in vitro*, decreased RANKL mRNA levels by 60%. Kusumi et al. (34) have similarly reported a decrease in RANKL mRNA expression and sRANKL release from human osteoblasts following 7% cyclic tensile strain; they also found that mechanical strain increased OPG synthesis. The present study builds on these findings, providing evidence for an upstream mechanism, and shows that contrary to what one might have expected, mechanical stress up-regulated the synthesis of IL-1β, TNF-α, and IL-6, three cytokines known to be potent stimulators of bone resorption *in vitro* (14–17).

IL-1β, TNF-α, and IL-6 have also been shown to stimulate osteoclast differentiation and bone resorption in a synergistic manner (35), and perhaps unexpectedly, to increase the production of both RANKL and OPG in the human osteosarcoma cell line MG-63 (36–38), although the dominant outcome was a net increase in RANKL activity (39, 40). We were therefore surprised to find that while intermittent tensile strain up-regulated IL-1β, TNF-α, and IL-6 synthesis, OPG production increased and sRANKL decreased, and when tested in an osteoclast resorption assay, culture supernatants from mechanically deformed cells were found to be inhibitory. This highlights the importance of bioassays. The bone literature contains a good deal of information about gene expression in normal and transformed cell lines, rather less about whether the expressed genes of interest are translated into protein, and if they are, whether the proteins are biologically active – in other words, real functional molecules.

We have previously shown that cyclic mechanical strain in the same model system inhibits IL-10 and stimulates IL-12 production by mouse calvarial osteoblasts (28), two cytokines with the ability to inhibit bone resorption. IL-10 selectively blocks osteoclastogenesis by inhibiting the differentiation of osteoclast progenitors into preosteoclasts (41, 42), while IL-12 inhibits RANKL-induced osteoclast formation in mouse bone marrow cell cultures, an effect mediated by IFN-γ (43, 44). IL-10 also suppresses osteoblast differentiation in mouse bone marrow cultures by inhibition of TGF-β1 production (45, 46).

These data underline the complexity of the biological response of osteoblasts to mechanical deformation and the potential disadvantage of investigating a relatively small number of cytokines at any one time. The fusion of real-time RT-PCR with microarray technology, which enables a large panel of genes to be screened at the same time under identical experimental conditions using relatively small quantities of RNA, provides an opportunity to significantly expand our knowledge of the number of mechanoresponsive genes expressed by bone cells. This has been used recently for periodontal ligament cells in an attempt to understand cell–cell signaling in terms of cytokine networks, and how these regulate complex biological processes such as tooth movement (47, 48). The downside is that more genomic data increases the difficulty of establishing a coherent sequence of events at the protein level.

This brings us to the significance of the present findings in the context of intact bone. Mechanical strain is an important determinant of bone mass and architecture, and the introduction of *in vivo* models in which carefully controlled external loads could be applied to bone, led to important advances in understanding the strain-dependent adaptation of bone to altered function (49–51). These showed that increased bone strains above a certain critical threshold resulted in bone formation, while reductions in strain magnitude resulted in bone loss and osteopenia. In the jaws, for example, masticatory hypofunction resulting from reduced occlusal loading leads to a reduction in alveolar bone mass and bone mineral density (52–55). Stress-shielding and disuse atrophy resulting from the implantation of rigid metallic devices into bone, is also a well-recognized complication of total hip arthroplasty and fracture fixation in orthopedic surgery (56–58).

To describe this tissue-level regulatory negative-feedback mechanism and add some clarity to the relationship between form and function in bone, the principle of a “mechanostat” for regulating bone mass was revived by Frost (59); the basic idea being that for each bone in the skeleton, there is a functional or mechanically adapted state within the boundaries of which normal bone mass is maintained. Osteoblasts cultured *in vitro* are deprived of the mechanical stimuli to which they would normally be exposed *in vivo* – in other words, the cells are in a physiological default state that in the intact skeleton leads to a decrease in bone strains below the critical threshold required for the maintenance of normal osseous architecture. The result is a localized negative skeletal balance or osteopenia and the reason why *in vitro* models are ideal for investigating bone resorption – the osteopenia is not permanent, however, and can be reversed by the restoration of normal functional loading.

The use of neonatal mouse calvaria as a source of primary cells of the osteoblast lineage as an alternative to transformed cell lines in bone biology is well-established. However, phenotypic differences exist between individual bones of the skeleton depending on their anatomical location, and calvarial and limb bones do not demonstrate the same responses to mechanical loading. Rawlinson et al. (60) recorded normal functional strains as low as 30 microstrain (μϵ) on rat parietal bone and found that unlike tibial osteoblasts (derived from lateral plate mesoderm), calvarial osteoblasts (of neural crest cell origin) did not show the same early responses to dynamic mechanical strain. Direct strain measurements in a human volunteer further showed that in the skull, the highest strains recorded (200 μϵ) were 10-fold lower than for the tibia (61), levels that in the rest of the skeleton would lead to profound bone loss.

Differences between neural crest and mesodermal bone in the concentration of growth factors (62, 63), heterogeneity of the enzymes produced by their osteoclasts (64, 65), patterns of expression of bone morphogenetic proteins (66) and the abundance of several matrix proteins, notably collagen in calvarial bone (67) have been reported. However, none provide an adequate answer to the question: what makes calvarial bone resistant to levels of mechanical strain that in the rest of the skeleton would lead to profound bone loss? It cannot be because calvarial bone is derived from the neural crest – the bones of the jaws are also of neural crest cell origin and do not show the same resistance to reduced mechanical loading. The well-characterized primary human calvarial and femoral osteoblasts now available from commercial sources provides an opportunity to further investigate these aspects of the mechanobiology of bone, but whether *in vitro* models are able to provide the answer remains to be seen.

In conclusion, the findings of this investigation are counterintuitive because IL-1β, TNF-α, and IL-6 have well-established reputations as bone resorptive agents. Nevertheless, they are pleiotropic molecules with multiple biological activities in addition to the stimulation of resorption, underlining the complexity of the biological response of osteoblasts to mechanical deformation, and the need to understand cell–cell signaling in terms of cytokine networks. It is also important to recognize that osteoblasts cultured *in vitro* are in a physiological default state that in the skeleton leads to decreased bone strains and osteopenia; this suggests that the application of mechanical strain to osteoblasts *in vitro* results in an osteogenic stimulus by restoring the metabolic activity of the cells to levels approaching that produced by functional osteoblasts *in vivo*.

## Conflict of Interest Statement

The authors declare that the research was conducted in the absence of any commercial or financial relationships that could be construed as a potential conflict of interest.
